# Assessing social competence and antisocial behaviors in children: item response theory analysis of the home and community social behavior scales

**DOI:** 10.1186/s40359-023-01045-1

**Published:** 2023-01-24

**Authors:** Silje Sommer Hukkelberg, Björn Andersson

**Affiliations:** 1 The Norwegian Center for Child Behavioral Development (NUBU), Essendropsgt 3, Majorstuen, Postbox 7053, 0306 Oslo, Norway; 2 Centre for Educational Measurement (CEMO), University of Oslo, Blindern, Postboks 1161, 0318 Oslo, Norway

**Keywords:** Children, Antisocial behavior, Social competence, HCSBS, Item response theory, Psychometrics

## Abstract

**Background:**

The Home and Community Social Behavior Scales (HCSBS) is a rating scale that assesses social competence and antisocial behavior among children and youths between ages 5–18. The present study aimed to investigate the psychometric properties of the HCSBS by applying item response theory (IRT).

**Methods:**

The HCSBS was completed by parents of 551 Norwegian children refereed to three independent interventions towards problem behaviors. Data used in this study was collected before the interventions started. IRT was carried out in R version 4.0.0 to investigate HCSBS items, subscales and main scales.

**Results:**

The results showed that the two-dimensional IRT models for social competence and antisocial behavior were the most appropriate. The measurement precision of the scales was high for a large range of the latent spectrum, and estimated reliabilities were satisfactory. Model evaluations indicated that the overall model fit for the scales were acceptable, but some misfit existed with respect to specific item pairs.

**Conclusion:**

The results indicate that the HCSBS is a reliable measurement instrument although there is still a potential for improvement by revising some of the items.

**Supplementary Information:**

The online version contains supplementary material available at 10.1186/s40359-023-01045-1.

## Background

Children and adolescents with behavioral problems show an increased risk for developing negative outcomes like school dropout, social exclusion, law breaking, psychopathology, and other health-related problems [1–3]. The costs are considerable, for each individual and for the society at large [4, 5]. Empirical findings show that child problem behaviors often operate in concert with poor social competence [6, 7]. This suggests that it may be advantageous to assess both concepts when children show high levels of resistant problem behaviors and are considered for intervention programs. However, in order to identify, monitor and treat behavioral problems and interpersonal challenges, reliable and validated screening instruments are required.

### Problem behavior and social competence

A growing body of research has demonstrated an inverse association between child conduct problems and social competence [8–11]. That is, children who display high levels of conduct problems tend to show low levels of social competence, or the other way round. Persistent child problem behaviors is a well-known risk factor associated with a troublesome future pathway that may follow into adolescents and sometimes into adulthood [12]. On the other hand, social competence is considered a protective factor associated with positive development and healthy functioning [13, 14] that e.g., facilitate school success [15–17]. Whereas child problem behavior is apparent through aggressive, oppositional or inattentive behaviors that may cause considerable challenges for parents, social competence is characterized by e.g., awareness about others needs and situation- appropriate behaviors [18]. Children with poor social competence can show inadequate social information processing and poor adaption to situations, which further result in rejection and dislike from peers and exclusion from social groups. Nevertheless, both problem behavior and social competence should be considered in relation to a child’s age, as both concepts are characteristically dynamic and complex in nature. Some have advocated that the two constructs should be considered separate albeit related dimensions of social functioning [19].

A considerable number of studies show that there is a significant association between child problem behavior and social competence, however, not all children with problem behavior are socially unskilled, or vice versa. It may, in fact, be difficult to disentangle the temporal precedence of the two concepts. Some findings suggest that poor social competence may contribute to maintain or increase the intensity of child problem behaviors [20], as it fosters frustration and limits a child’s possibilities to develop positive interactions with others. In addition, it should be noted that the strength between problem behavior and social competence varies considerable across studies, and depend on whether the two concepts are assessed by the same respondents and the bandwidth of the constructs being measured. A recent meta-study based on 54 independent studies among children aged 3–13, showed an overall negative and significant correlation of medium effect size (*r* =  − 0.42) between behavioral problem and social competence [21]. The magnitude was even higher (*r* =  − 0.47, *p* < 0.001) when both constructs were assessed by parents, but considerable lower when one of the constructs were reported by a parent and the other by a teacher (*r* =  − 0.17, *p* < 0.001). However, several of the studies relied on different measurement instruments to assess child problem behavior and social competence, and some date back to the 1980s and early 1990s, and have shown varying psychometric properties [22]. Overall, these findings suggest that it could be worthwhile to consider using instruments that are newer and more psychometrically sound, and that address both social competence and problem behavior. This would probably ease use and interpretation of both concepts.

### The home and community social behavior scales

The Home and Community Social Behavior Scales (HCSBS [23]) is a parent-reported instrument that provides insight into children’s Social Competence (Scale A) and Antisocial Behavior (Scale B) using the same rating scale. The HCSBS scale is the home version of the School Social Behavior Scales (SSBS [24]). Whereas Scale A assesses the big picture of adaptive and positive social behaviors, Scale B measures the broad concept of socially related problem behaviors. Both scales comprise two subscales. The social competence scale measures Peer relations (e.g., assesses positive skills with peers) and Self-management/compliance (e.g., responses to adult expectations), whereas the Antisocial behavior scale includes the Antisocial/aggressive scale (e.g., overt violation, intimidation or harm to others) and the Disruptive/demanding scale (e.g., behaviors likely to disrupt ongoing activities and place inappropriate demands on others). Validation studies indicate that the HCSBS has good psychometric properties [25–27], in addition, two systematic reviews evaluating the Scale A have encouraged the use of the HCSBS to assess social competence [28, 29]. However, a drawback pertaining these studies is that they rely on classical test theory that e.g., treats the ordinal item scores as continuous, and puts emphasis on total scores where all items are equally weighted [30]. Although some studies have considered the social competence scale[31, 32], few studies have evaluated the dimensionality of the HCSBS or its parallel teacher form (SSBS). In the present study we aimed to extend previous knowledge about the HCSBS by applying item response theory (IRT), which is the preferred method for psychometric evaluations [33, 34].

### Item response theory

Item response theory (IRT) comprises a family of flexible statistical models and techniques that can be used to evaluate the psychometric properties of a scale, improve scoring accuracy, increase measurement precision, and form the basis for evaluating item properties [35, 36]. In this study, we use polytomous IRT [37, 38] to model the response options for the ordinal observed variables of the HCSBS. Like classical test theory, IRT assumes that the latent construct cannot be measured directly, but instead is measured by a set of indicators. IRT, however, specifies a non-linear relationship between the continuous latent variable and the categorical observed variables. IRT models are typically characterized by the two parameters discrimination parameters and location parameters [39]. The discrimination parameters indicate how well the items can differentiate between participants with different values of the latent variable (similar to an index of item discrimination), while the location parameters inform about the average value of the latent variable associated with a particular response category (similar to item difficulty). With basis in the estimated item parameters, IRT allows for a detailed description of item and scale properties.

### The present study

In this study, we utilized IRT to investigate three research questions. First, we investigate dimensionality of the Social competence (Scale A) and the Antisocial behavior (Scale B) scales as measured by HCSBS, by comparing one-dimensional models with two-dimensional models for each scale. For the two-dimensional models, the different subscales of Scale A and Scale B were considered as measuring separate but correlated dimensions of social competence and antisocial behavior, respectively. We evaluate the estimated models with respect to the model fit and item fit. Second, we infer the item and scale properties for the subscales Peer relations (Scale A1) and Self-management/compliance (Scale A2), and Antisocial/aggressive behavior (Scale B1) and Defiant/disruptive behavior (Scale B2), using item category probability curves, item information functions, and test information functions. Third, we estimate IRT model-based reliability indices for sum scores and IRT scores to evaluate the precision of measurement of the scale scores.

The benefits of IRT are a more realistic model for item responses (which accounts for the ordinal nature of the item scores), improved evaluation of item properties, increased measurement precision through pattern scoring, full-information methods for estimation, and access to detailed tools to evaluate scale properties such as scale difficulty via test characteristic curves and scale precision from test information functions. To our knowledge, this is the first study to evaluate the HCSBS using IRT and as such the study provides important additional information on the properties of the HCSBS, which is useful for practitioners and researchers alike.

## Method

### Participants

Data derived from 551 children who were enrolled into three independent interventions aimed to reduce child conduct problems [40–42]. The present data was collected before the interventions started. A parent or a primary caretaker completed the HCSBS questionnaire [23]. The eligible families agreed to participate in the study by signing a written informed consent document. In line with standard procedures in the Norwegian children’s services, the interventions were offered based on practitioners’ clinical judgments rather than formal screening. In all samples, children were excluded from participation if they were diagnosed with autism, had been exposed to sexual assaults, or were intellectually disabled, or if parents had serious mental health problems. The Regional Committee for medical research ethics approved all three studies (REK 2.2006.2066).

Sample 1 consisted of children from 137 families, recruited from 11 agencies situated in different municipalities in Norway. Children were between the ages of 3 and 12 (*M* = 8.56, *SD* = 2.35) and 50 (36.5%) were girls. Parents were on average 37.42 years (*SD* = 6.34). Among the participating children, 66 (48.2%) lived with both biological parents, 21 (15.3%), and 50 (36.5%) lived with single parents. The average gross annual family income was 509,610 Norwegian Kroner (*SD* = 347.70), which is approximately $51,873, representing an upper middle income level. According to parent self-report, 37 (27%) had a college or higher university degree, 83 (60.6%) had finished high school, and 17 (12.4%) had completed junior high school or elementary school. Most of the parents had Norwegian background (126 or 92%), only one (0.7%) was from another western European country, and 8 (7.3%) reported “other” ethnicity. Sample 2 included children from 198 families recruited from 9 municipalities in Norway. Child age ranged from 3 to 12 years (M = 7.64, SD = 2.19), and 39 (19.7%) were girls. The average age of the reporting parent was 36.30 years (SD = 6.07). Among the participating children, 106 (53.5%) lived with both biological parents, 29 (14.6%) with a parent cohabiting with another adult, and 63 (31.8%) lived with single parents. The average family income was 564.090 Norwegian Kroner (SD = 267.05), which is approximately $ 57,469 and representing an upper middle income level. Parent self-report showed that 80 (40%) had a college or higher university degree, 96 (48.5%) had finished high school, and 22 (11.1%) had completed junior high school or elementary school. Most parents reported a Norwegian background (182 or 92%), and 16 (7.2%) reported “other” ethnicity.

Sample 3 included children from 216 families from five municipalities in Norway. One child was excluded because of an autism diagnosis. Child age ranged from 3 to 12 years (M = 7.28, SD = 2.61), and 69 (31.9%) were girls. The average age of the parents was 35.31 years (SD = 6.08). Hundred and ten children (50.9%) lived with both biological parents, 27 (12.5%) with a parent cohabiting with another adult, and 79 (36.6%) lived with single parents. The average gross annual family income was 539.110 Norwegian Kroner (SD = 328.29), which is approximately $54, 827 and represents an upper middle income level. Among the parents, 85 (39.4%) had a college or higher university degree, 114 (52.8%) had finished high school, and 17 (7.9%) had completed junior high school or elementary school. Most of the parents had a Norwegian background (202 or 93.5%), four (1.9%) were from other western European countries, and the remaining 10 (4.6%) reported “other” ethnicity.

### Instrument

The Home and Community Social Behavior Scales (HCSBS) [23] is a screening instrument that assesses social competence (Scale A) and antisocial behavior (Scale B), which each comprises 32 items rated on a 5-point scale from 0 (never) to 4 (frequently). Scale A consists of the subscales Peer relations (A1; 17 items) and self-management/compliance (A2; 15 items) and includes evaluations of items like “completes chores without being reminded”, “shows self-control”, and “is invited by peers to join in activities”. Higher scores indicate higher levels of social competence. Scale B measures antisocial behavior and contains the two dimensions Antisocial/aggressive (B1; 17 items) and defiant/disruptive (B2; 15 items) behaviors. Items include e.g., “gets into fights”, “bothers and annoys others”, and “acts impulsively without thinking”. Higher scores on this scale indicate more antisocial behavior. The HCSBS was translated from English to Norwegian by the Norwegian Center for Child Behavioral Development.

### Statistical analysis

We used the statistical programming environment R Version 4.0.0 [43] and the package mirt [44] to estimate the IRT models and evaluate item and scale properties.

*Item response theory (IRT) analysis* We estimated unidimensional and multidimensional graded response models (GRM) [38] and generalized partial credit models (GPCM) [37] for the ordinal observed variables using marginal maximum likelihood estimation [45]. The functional form of the IRT model was decided based on the AIC [46]. To characterize the properties of the items on the scales, we examined item category probability curves and item information functions [47]. To assess the measurement precision of the scales for different values of the latent variable, we computed scale information functions [48].

*Dimensionality* We evaluated the dimensionality of each of the scales (A and B) by fitting a series of models. First, we specified unidimensional models for each of the subscales Peer relations (17 items, Scale A1), self-management/compliance (15 items, Scale A2), Antisocial/aggressive (17 items, Scale B1) and defiant/disruptive (15 items, Scale B2). If the unidimensional models for each subscale were considered acceptable, we specified models for all the items in Scales A and B, respectively. Hence, for the items in Scale A, we specified (1) a unidimensional model and (2) a two-dimensional model where the correlated latent variables were related to the Peer relations or self-management/compliance items. For the items in Scale B, we specified (1) a unidimensional model and (2) a two-dimensional model where the correlated latent variables were related to Antisocial/aggressive or defiant/disruptive items. For either scale, we compared the model fit between the unidimensional and two-dimensional models using likelihood ratio tests.

*Model evaluation* Model fit was assessed via the limited-information fit statistic M2 [49], with the fit statistics root mean square error of approximation (RMSEA_2_) and the standardized root mean square residuals (SRMSR), where RMSEA_2_ ≤ 0.089 and SRMSR < 0.05 indicated good approximate model fit [50], and with residual analysis [51]. Residual correlations larger than 0.1 in absolute value were further investigated with respect to the content of the corresponding pairs of items. Fit of individual items was evaluated using $S - \chi^{2}$ item fit statistics [52, 53].

*Reliability estimation* The reliability of the sum scores were estimated using Monte-Carlo computer simulations [54]. We also estimated the reliability of expected á posteriori (EAP) IRT scores with the approach defined in Kim [55].

## Results

### Descriptive statistics

The mean of Scale A1 was 41.52 (SD = 12.16, range: 4–68) whereas Scale A2 had mean 31.96 (SD = 9.81, range: 7–59). The mean of Scale B1 was 22.04 (SD = 10.66, range: 0–56) and Scale B2 had mean 22.37 (SD = 10.86, range: 0–57). The total scales showed means of 73.21 (SD = 20.14, range 16–127) and 44.41 (SD = 20.8, range 3–108), for Scale A and B, respectively. The total scores correlated *r* =  − 0.59 (*p* < 0.001). Additional file 1: Tables S1 and S2 provide item means (SD) and item-total correlations for Scales A and B. By convention, inter-item correlations equal or below 0.4 indicate very good discrimination. Only one item of Scale A showed poor discrimination (item 17) while three items were below 0.4 for Scale B (items 2, 13, and 32). Based on the content of the items, we still included them in the subsequent analysis.

### IRT model selection and model evaluation

The model selection resulted in the GRM being preferred over the GPCM for all subscales. A series of IRT models were then estimated to investigate the assumptions of one-dimensional scales, and an overarching one- or two-dimensional model within social competence and antisocial behavior, respectively. These results are presented in Table 1, showing that scales A1 and B2 had good approximate fit according to the RMSEA_2_ (0.080 and 0.071 ≤ 0.089) but some lack of fit according to the SRMSR (0.069 and 0.082 > 0.05). Meanwhile, A2 and B1 showed evidence of some misfit according to both the RMSEA_2_ (0.109 and 0.108 > 0.089) and the SRMSR (0.071 and 0.074 > 0.05). However, we judged the fit of the unidimensional models for each subscale to be sufficiently good to pursue further analyses. Regarding the models for the total scales of social competence (scale A) and antisocial behavior (scale B), scale A showed significantly better model fit as a two-dimensional model rather than a one-dimensional model ($\chi^{2} (df)$: 609.172 (1), *p* < 0.001). The correlation between the two latent variables representing Peer relations and Self-management/compliance was estimated to be 0.725 (95% CI 0.675, 0.775). The model fit for the two-dimensional model showed good approximate fit with respect to the RMSEA_2_ (0.073 ≤ 0.089) but some misfit considering the SRMSR (0.077 > 0.05). For scale B, the two-dimensional model also fitted better than the one-dimensional model ($\chi^{2} (df)$: 6.869 (1), *p* = 0.009) but the correlation between the latent variables representing Antisocial/aggressive and Defiant/disruptive was estimated to be very high at 0.979, with 95% CI (0.962, 0.995). The model fit of the two-dimensional model showed good approximate fit with respect to the RMSEA_2_ (0.088 ≤ 0.089) but some misfit with the SRMSR (0.079 > 0.05). The item fit statistics are included in Additional file 1: Tables S3 and S4. Two items on each scale had significant misfit at a significance level of 0.05: items 4 ($S - \chi^{2} (df)$ = 131.5 (106); *p* = 0.047), and 9 ($S - \chi^{2} (df)$ = 161.1 (128); *p* = 0.025) of Scale A and items 9 ($S - \chi^{2} (df)$ = 149.1 (122); *p* = 0.048) and 25 ($S - \chi^{2} (df)$ = 129.013 (103); *p* = 0.042) of Scale B. After adjusting the significance level with the Bonferroni-Holm correction [56], none of the items displayed a lack of fit and we concluded that the item fit was acceptable.

**Table 1:** Model fit statistics for unidimensional and two-dimensional models

Scale/models	BIC	M_2_ (df)	*p*	RMSEA	(95% CI)	SRMSR
Peer relations (Scale A1, 1D)	24,186.692	304.54 (68)	< 0.001	0.080	(0.069; 0.090)	0.069
Self-manag/compliance (Scale A2, 1D)	21,447.134	337.62 (45)	< 0.001	0.109	(0.096; 0.122)	0.071
Antisocial/aggressive (Scale B1, 1D)	23,069.409	502.651 (68)	< 0.001	0.108	(0.097; 0.118)	0.074
Defiant/disruptive (B2, 1D)	21,345.987	171.017 (45)	< 0.001	0.071	(0.058; 0.085)	0.082
Social competence (1D)	45,941.976	1624.549 (368)	< 0.001	0.079	(0.075; 0.083)	0.085
Antisocial behavior (1D)	43,640.189	1944.889 (368)	< 0.001	0.088	(0.084; 0.092)	0.079
Social competence (2D)	45,339.114	1453.484 (367)	< 0.001	0.073	(0.069; 0.078)	0.077
Antisocial behavior (2D)	43,639.63	1932.957 (367)	< 0.001	0.088	(0.083; 0.093)	0.079

Scale A assesses social competence (A1: peer relations, A2: self-management/compliance), scale B assesses antisocial behavior (B1: antisocial/aggressive, B2: defiant/disruptive). In IRT, M_2_ (Maydeu-Olivares and Joe 2005) estimates global model-data fit

1D, one-dimensional; 2D, two-dimensional; BIC, Bayesian Information Criterion; df, degrees of freedom; RMSEA, root mean square error of approximation; SRMSR, standardized root mean square residual

The estimated reliabilities for each scale are presented in Table 2. The reliability of the sum scores for A1 was estimated to be 0.920 and the reliability for IRT scores 0.928, whereas the sum scores for A2 was estimated to be 0.894 and the reliability for IRT scores 0.912. Considering antisocial behaviors, the estimated reliability for B1 was 0.887, and for the IRT scores 0.939. B2 had estimated sum score reliability 0.902 and IRT score reliability 0.942. Overall, the results indicate that both scales are highly reliable.

**Table 2:** Reliability coefficient estimates

	Social competence (Scale A)			Antisocial behavior (Scale B)		
	Total scale	Peer relations	Self-management/compliance	Total scale	Antisocial/aggressive	Defiant/disruptive
Sum scores	0.945	0.920	0.894	0.944	0.887	0.902
IRT EAP scores	–	0.928	0.912	–	0.939	0.942

EAP, expected á posteriori; IRT, item response theory

### Item and scale properties

**Fig. 1:**
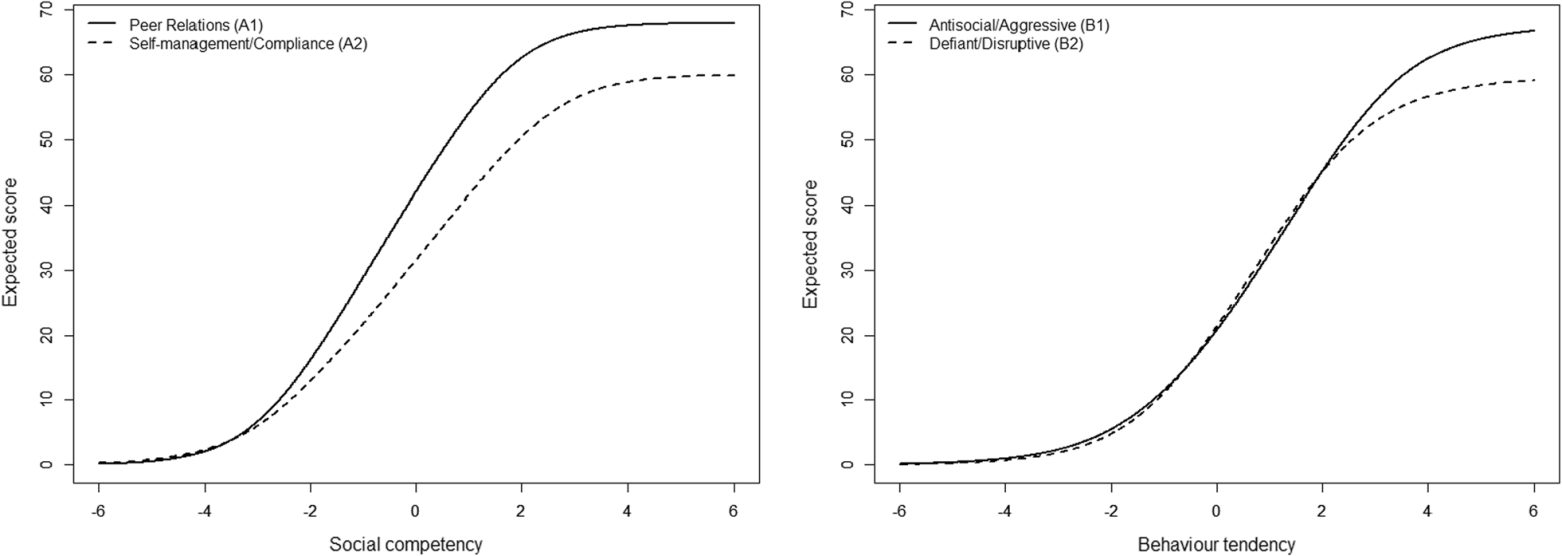
Expected scale score functions

Figure 1 shows the expected scale score functions for the subscales Social competence and Antisocial behavior, respectively. The results show that the scales are well-matched relative to the population under study, with very few respondents expected to score at the minimum or maximum of the respective scales.

**Fig. 2:**
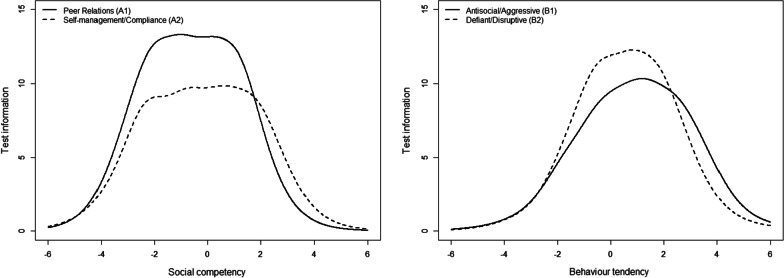
Scale information functions

**Fig. 3:**
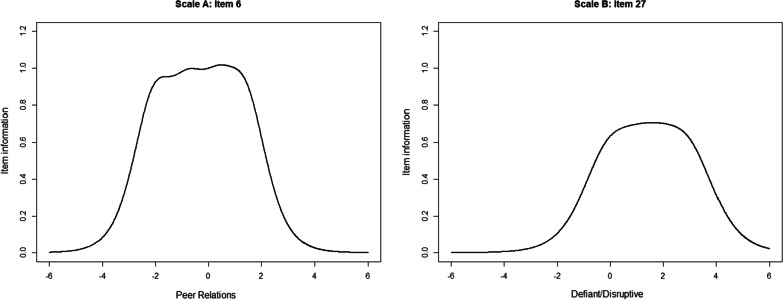
Information functions for item 6 of scale A and item 27 of scale B

To illustrate the measurement precision of the scale scores based on the fitted two-dimensional model, we plotted the scale information functions for each of the subscales (Fig. 2). Scale information functions are the sum of the item information functions and indicate the precision of the scale as a whole for different values of the underlying latent variable [57]. Generally speaking, the scale information functions indicate that both scales have a high measurement precision across the latent spectrum, with peaks around or higher than 10 and lows that are above 5 for the most relevant ranges of the latent spectrum. The social competence scales have peaks at the middle range of the latent spectrum, and both scales provide the highest measurement precision for values of the latent variable between − 2 and 2, which is expected to cover 95% of the respondents. On the other hand, both of the subscales of antisocial behavior have somewhat higher measurement precision for higher values of the latent variables, and the Antisocial/aggressive scale provides the most information between − 1 and 2, whereas the Defiant/disruptive scale provides the most information between 0 and 3. Much of the same pattern occurred at the item-level (Additional file 1: Figs. S3 and S4), as illustrated in Fig. 3 by item 6 (“understands problems ”, scale A1) and item 27 (“boasts”, scale B2).

### Residual analysis

We conducted detailed analysis of the residual correlation matrix to identify sources of misfit in the model. Table 3 shows the highest residual correlations within each of the four scales. Residual correlations for the Peer relation scale involves two items that show overlap in content (empathy; items 6 and 22), whereas in the Self-management/compliance scale we found two items with the same wording (completes chores; item 13 and 14). Considering the Antisocial/aggressive scale, substantial residual correlations were found for item 5 and item 25 that both reflect aggressive behavior, and item 6 and 28 that reflect dishonesty. Lastly, the residual correlations in the Defiant/disruptive scale reflect overlap in content (headstrong; item 2 and 13) and highlighting oneself (item 3 and 20, and item 7 and 27).

**Table 3:** The highest residual correlations within each of the scales

	Residual correlation
*A1: Peer relations*	
#22 sensitive to feelings * #30 invited by friends	0.15
#6 understands problems * #30 invited by friends	0.14
#6 understands problems * #22 sensitive to feelings	0.14
*A2: Self-management/compliance*	
#13 completes tasks * #14 completes tasks on time	0.20
#17 behaves at school * #18 asks for help	0.19
#23 responds appropriately * #24 controls temper	0.17
#24 controls temper * #31 shows self-control	0.17
*B1: Antisocial/aggressive scale*	
#5fights * #25 trouble	0.26
#6 is dishonest * #28 not trustworthy	0.19
#5 fights * #28 not trustworthy	0.17
*B2: Defiant/disruptive*	
#3 defiant * #20 insults friends	0.21
#2 takes things * #13 not share	0.21
#7 teases * #23 difficult to control	0.21
	0.19
#7 teases * #27 boasts	0.18

## Discussion

In the present study, we applied IRT to investigate the psychometric properties of the social competence and Antisocial behavior scales in the HCSBS. To our knowledge this is the first study to use IRT to examine the HCSBS. Both social competence and antisocial behavior have received extensive attention in the literature on child development and adjustment, but to advance knowledge about these constructs, valid and reliable instruments are required. Our results showed acceptable model-fit for the two-dimensional structure of social competence, and Peer relations and Self-management/compliance appear to measure different but related aspects of social competence. Considering the Antisocial behavior scale, results showed a strong correlation between the Antisocial/aggressive and Defiant/disruptive scales, suggesting that the two concepts can not empirically be considered distinct from each other. Reliabilities for Social competence and Antisocial behavior subscales and total scale-scores were high. Furthermore, our analysis revealed that individual items varied in terms of how much information they contributed to the overall measurement precision of the scale. The scales also had different properties in terms of measurement precision. Peer relations showed higher test information across the latent spectrum than the Self-management/compliance scale, whereas the Defiant/disruptive scale had higher overall test information compared to the Antisocial/aggressive scale. All scales showed most information around the middle of the latent spectrum and can thus be viewed as well-matched with the intended target population. Inspections of residual correlations indicated that several items, in both scales, should be further evaluated and potentially revised in future versions of the scale.

The study has several implications for research and practice. First, the HCSBS is one of few scales that measures problem behavior and social comptence using the same rating scale. This makes it easier to use and interpret the relation between the two concepts. Our findings from a sub-clinical sample of Norwegian children indicate that interpretations based on the subscale scores of the HCSBS is supported, and that the sum scores and IRT scores of the subscales are highly reliable. However, practitioners should be aware that the HCSBS does not distinguish well between Antisocial/aggressive and Defiant/disruptive behaviors. Our item information analyses showed that the social competence and antisocial behaviors scales were most informative around the mean of the latent spectrum. As a result, the scales are well-matched with the target population, but do not very precisely assess respondents with very low or very high values. Thus, it seems most appropriate to measure antisocial behavior based on the combined scores from both subscales. The two scales measuring social competence do however provide distinct and separate scoring. It should be noted, that although there is broad consensus that social competence comprises interpersonal behaviors associated with a positive social outcome [58, 59], the indicators vary considerably [60, 61]. Last, it is unknown whether the combination of poor social competence and antisocial behavior is predictive of more serious child problems compared to other factors. This should be examined further. Several limitations apply to this study. First, our sample consisted of a low number of girls (*n* = 157), which made it impossible to consider gender differences. Findings indicate gender differences in both social competence and antisocial behavior [13, 62], thus such analyses would provide valuable information. Furthermore, our results are based on a sub-clinical sample of children, and do not necessarily generalize to other samples. It is also unclear if the misfit we identified for some items relates to the Norwegian version only, so more studies on other translations are warranted. Modifications of specific items would however improve the instrument further.

## Conclusion

The findings of the present study add new knowledge about the psychometric properties of HCSBS. Our results indicate that the HCSBS has clinical utility when it comes to measure social competence and antisocial behavior in children. Thus, practitioners can rely on one instrument only instead of using different instruments to measure and monitor social competence and antisocial behavior in children with social and behavioral difficulties.

## Supplementary Information

**Additional file 1: Table S1**. Descriptive statistics for the social competence (Scale A). **Table S2**. Descriptive statistics for the antisocial scale (Scale B). **Table S3**. Item fit statistics for scale A. **Table S4**. Item fit statistics for scale B. **Figure S1**. Item category response functions for Scale A. **Figure S2**. Item category response functions for scale B. **Figure S3**. Item information functions for scale A. **Figure S4**. Item information functions for Scale B.

## Data Availability

The data that support the findings of this study are not publicly available as they are part of a because of the sensitivity of the information. Data are however available from the local research committee (post@nubu.no) or the corresponding author upon reasonable request.
