# Supplementary material for: Assessing social competence and antisocial behaviors in children: item response theory analysis of the home and community social behavior scales
Source: BMC Psychol. 2023 Jan 24;11:19. doi: 10.1186/s40359-023-01045-1 (PMC9875445; doi:10.1186/s40359-023-01045-1)
Supplement: Supplementary file 1 — Additional file 1: Table S1. Descriptive statistics for the social competence (Scale A). Table S2. Descriptive statistics for the antisocial scale (Scale B). Table S3. Item fit statistics for scale A. Table S4. Item fit statistics for scale B. Figure S1. Item category response functions for Scale A. Figure S2. Item category response functions for scale B. Figure S3. Item information functions for scale A. Figure S4. Item information functions for Scale B. [file 40359_2023_1045_MOESM1_ESM.docx]

| Item | | | *M* (SD) | Item-scale correlation |
| --- | --- | --- | --- | --- |
| A1: Peer Relations (17 items) | | |  |  |
| 1 | cooperates with friends | 2.67 (1.04) | | .61 |
| 4 | help friends | 2.87 (1.06) | | .59 |
| 5 | participates in family | 2.69 (1.02) | | .53 |
| 6 | understands problems | 2.18 (1.15) | | .66 |
| 9 | invites friends | 2.68 (1.15) | | .55 |
| 11 | skills | 2.19 (1.16) | | .63 |
| 12 | accepting | 2.72 (1.00) | | .60 |
| 15 | give-in | 2.03 (1.07) | | .50 |
| 19 | interacts with friends | 2.48 (1.20) | | .57 |
| 21 | initiating conversations | 2.69 (1.17) | | .62 |
| 22 | sensitive to feelings | 2.55 (1.12) | | .49 |
| 25 | on-going activities | 2.30 (1.07) | | .65 |
| 26 | leadership | 2.17 (1.13) | | .58 |
| 28 | compliments | 2.26 (1.13) | | .57 |
| 29 | assertive | 2.17 (.94) | | .57 |
| 30 | invited by friends | 2.54 (1.14) | | .67 |
| 32 | “looked up to” | 2.30 (1.14) | | .69 |
| A2 : Self-management/Compliance (15 items)  A2: Self-Management/Compliance | | |  |  |
| 2 | transitions | 2.47 (1.14) | | .42 |
| 3 | completes tasks | 1.51 (.98) | | .50 |
| 7 | calm | 1.84 (1.09) | | .51 |
| 8 | directions | 1.92 (.93) | | .62 |
| 10 | asks appropriately | 1.83 (1.13) | | .52 |
| 13 | completes tasks | 2.15 (1.08) | | .63 |
| 14 | completes tasks on time | 1.90 (1.05) | | .62 |
| 16 | follows rules | 2.31 (1.01) | | .65 |
| 17 | behaves at school | 2.63 (1.17) | | .37 |
| 18 | asks for help | 2.61 (.99) | | .55 |
| 20 | produces work | 2.85 (1.07) | | .52 |
| 23 | responds appropriately | 1.97 (1.00) | | .59 |
| 24 | controls temper | 1.47 (1.09) | | .54 |
| 27 | adjusts | 2.12 (1.00) | | .64 |
| 31 | self-control | 2.13 (1.06) | | .58 |

**Table S1***Descriptive statistics for the social competence (Scale A)*

*Note.* All variables had a range from 0 to 4. The item numbering corresponds to the HCSBS, and descriptions reflect part of the content.

**Table S2** *Descriptive statistics for the antisocial scale (Scale B)*

| Item | | | | *M* (SD) | Item-scale correlation |
| --- | --- | --- | --- | --- | --- |
| B1: Antisocial/Aggressive (17 items) | | | |  |  |
| 1 | blames others | | 2.29 (1.17) | | .45 |
| 4 | cheats | | 1.24 (1.13) | | .45 |
| 5 | fights | | 0.82 (1.01) | | .50 |
| 6 | dishonest | | 1.19 (1.11) | | .61 |
| 9 | easily triggered | | 2.33 (1.28) | | .59 |
| 11 | better | | 1.03 (1.10) | | .50 |
| 12 | destroys | | 0.59 (.89) | | .50 |
| 15 | disregards feelings | | 1.52 (1.03) | | .52 |
| 19 | aggressive | | 1.11 (1.13) | | .55 |
| 21 | whines | | 1.80 (1.22) | | .43 |
| 22 | argues with friends | | 1.49 (.93) | | .54 |
| 25 | trouble | | 0.99 (1.11) | | .58 |
| 26 | on-going activities | | 1.40 (1.09) | | .62 |
| 28 | not trustworthy | | 1.01 (1.12) | | .54 |
| 29 | cruel | | 0.25 (.67) | | .44 |
| 30 | acts spontaneously | | 1.94 (1.25) | | .54 |
| 32 | help | | 1.04 (.88) | | .36 |
| B2: Defiant/Disruptive (15 items)  **Defiant/Disruptive** | | |  | |  |
| 2 | takes things | | 1.04 (1.13) | | .34 |
| 3 | defiant | | 2.55 (1.11) | | .57 |
| 7 | teases | | 1.18 (1.03) | | .51 |
| 8 | disrespectful | | 1.60 (1.23) | | .71 |
| 10 | ignores | | 1.79 (1.21) | | .62 |
| 13 | not share | | 1.16 (1.02) | | .35 |
| 14 | temper outbursts | | 2.10 (1.26) | | .57 |
| 16 | attention | | 1.83 (1.32) | | .51 |
| 17 | threatens | | 1.09 (1.17) | | .64 |
| 18 | swears | | 1.16 (1.27) | | .54 |
| 20 | | insults friends | 1.01 (.94) | | .55 |
| 23 | difficult to control | | 1.75 (1.14) | | .70 |
| 24 | bothers | | 1.08 (1.00) | | .66 |
| 27 | boasts | | 0.89 (1.08) | | .51 |
| 31 | irritated | | 2.15 (1.20) | | .67 |

*Note.* All variables had a range from 0 to 4. The item numbering corresponds to the HCSBS, and descriptions reflect part of the content.

| Item # | S-X2 | df | p-value |
| --- | --- | --- | --- |
| 1 | 90.294 | 102 | 0.790 |
| 4 | 131.498 | 106 | **0.047** |
| 5 | 125.459 | 114 | 0.218 |
| 6 | 132.665 | 112 | 0.089 |
| 9 | 161.113 | 128 | **0.025** |
| 11 | 110.655 | 130 | 0.889 |
| 12 | 106.901 | 104 | 0.403 |
| 15 | 103.058 | 122 | 0.892 |
| 19 | 131.569 | 124 | 0.304 |
| 21 | 128.277 | 117 | 0.224 |
| 22 | 133.779 | 136 | 0.538 |
| 25 | 124.002 | 102 | 0.068 |
| 26 | 134.575 | 133 | 0.446 |
| 28 | 131.591 | 123 | 0.282 |
| 29 | 77.011 | 94 | 0.899 |
| 30 | 104.152 | 111 | 0.664 |
| 32 | 109.671 | 103 | 0.308 |
| 2 | 118.209 | 140 | 0.909 |
| 3 | 131.491 | 115 | 0.139 |
| 7 | 126.388 | 126 | 0.474 |
| 8 | 93.052 | 89 | 0.364 |
| 10 | 117.03 | 130 | 0.786 |
| 13 | 110.088 | 116 | 0.637 |
| 14 | 124.928 | 104 | 0.079 |
| 16 | 104.057 | 99 | 0.344 |
| 17 | 149.986 | 143 | 0.328 |
| 18 | 136.746 | 115 | 0.081 |
| 20 | 140.162 | 115 | 0.055 |
| 23 | 100.171 | 98 | 0.420 |
| 24 | 112.912 | 123 | 0.732 |
| 27 | 106.144 | 98 | 0.270 |
| 31 | 105.014 | 107 | 0.536 |

**Table S3.** *Item fit statistics for scale A*

| Item | S-X2 | df | p-value |
| --- | --- | --- | --- |
| 1 | 136.767 | 144 | 0.653 |
| 4 | 141.972 | 142 | 0.485 |
| 5 | 95.348 | 97 | 0.528 |
| 6 | 124.9 | 117 | 0.292 |
| 9 | 149.131 | 122 | **0.048** |
| 11 | 128.681 | 112 | 0.134 |
| 12 | 100.261 | 80 | 0.062 |
| 15 | 122.39 | 107 | 0.147 |
| 19 | 106.342 | 103 | 0.391 |
| 21 | 158.125 | 145 | 0.215 |
| 22 | 109.379 | 97 | 0.184 |
| 25 | 129.013 | 103 | **0.042** |
| 26 | 105.493 | 114 | 0.703 |
| 28 | 113.79 | 93 | 0.071 |
| 29 | 38.753 | 38 | 0.436 |
| 30 | 164.192 | 144 | 0.120 |
| 32 | 132.071 | 107 | 0.050 |
| 2 | 145.852 | 135 | 0.247 |
| 3 | 145.626 | 124 | 0.090 |
| 7 | 107.735 | 100 | 0.281 |
| 8 | 114.299 | 98 | 0.125 |
| 10 | 131.903 | 132 | 0.486 |
| 13 | 118.832 | 117 | 0.435 |
| 14 | 125.129 | 137 | 0.758 |
| 16 | 147.75 | 150 | 0.537 |
| 17 | 119.359 | 103 | 0.129 |
| 18 | 125.297 | 124 | 0.451 |
| 20 | 99.861 | 88 | 0.182 |
| 23 | 76.74 | 100 | 0.959 |
| 24 | 73.03 | 82 | 0.750 |
| 27 | 103.284 | 101 | 0.418 |
| 31 | 102.522 | 115 | 0.791 |

**Table S4.** *Item fit statistics for scale B*

**Figure S1**
 *Item category response functions for Scale A*


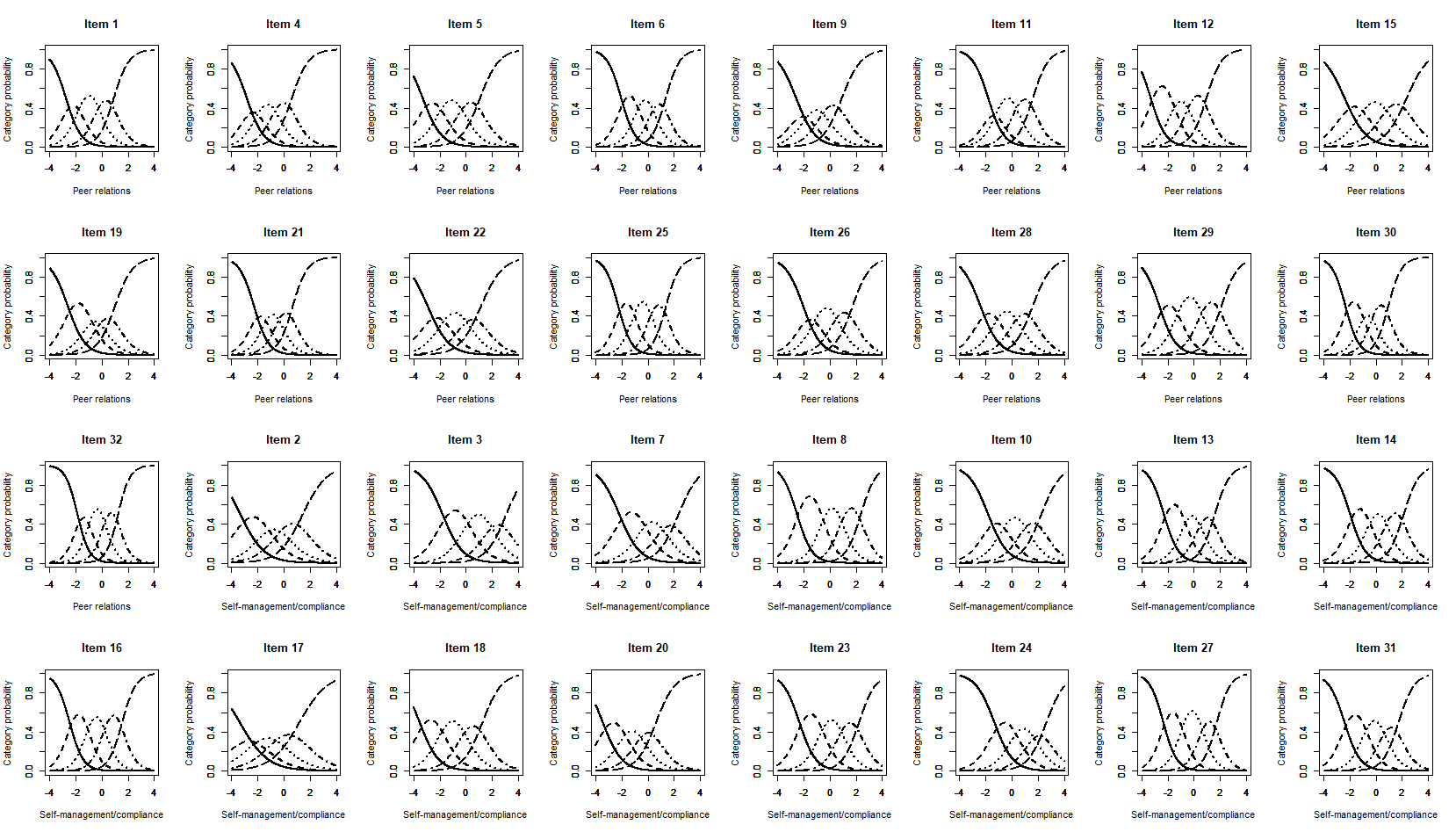


**Figure S2** *Item category response functions for scale B*

**
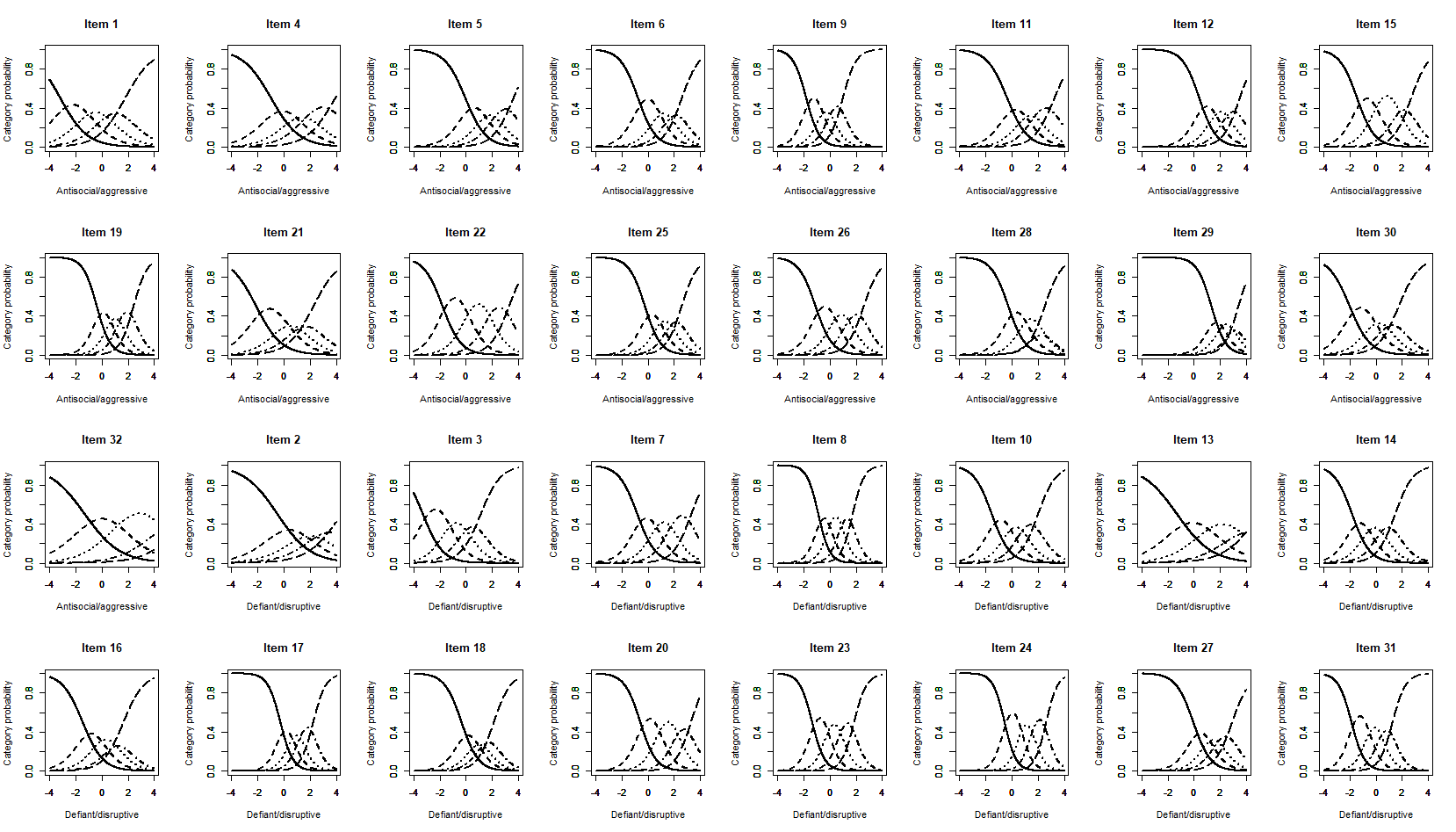
**

**Figure S3**

*Item information functions for scale A*

**
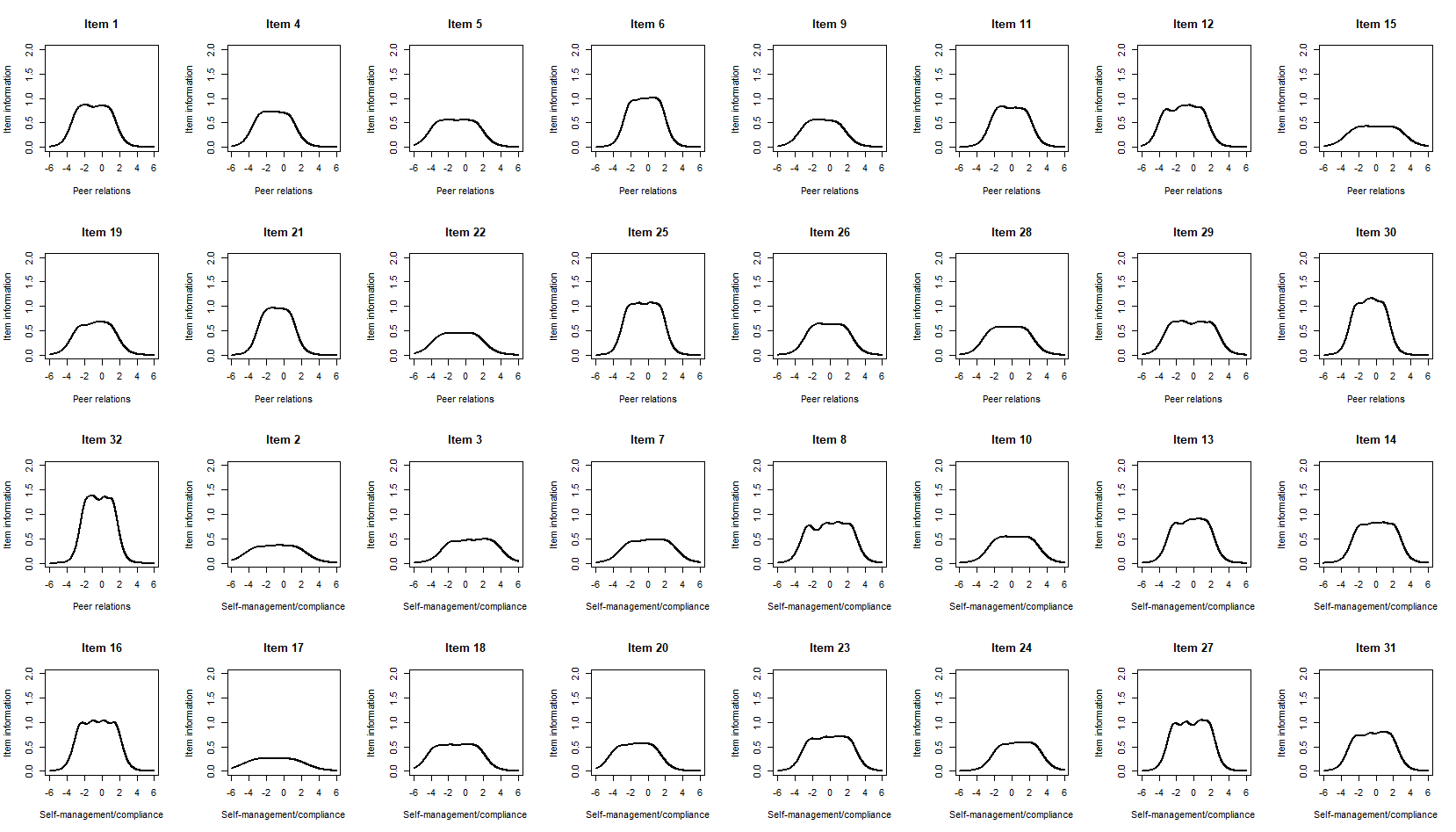
**

**Figure S4**

*Item information functions for Scale B*

**
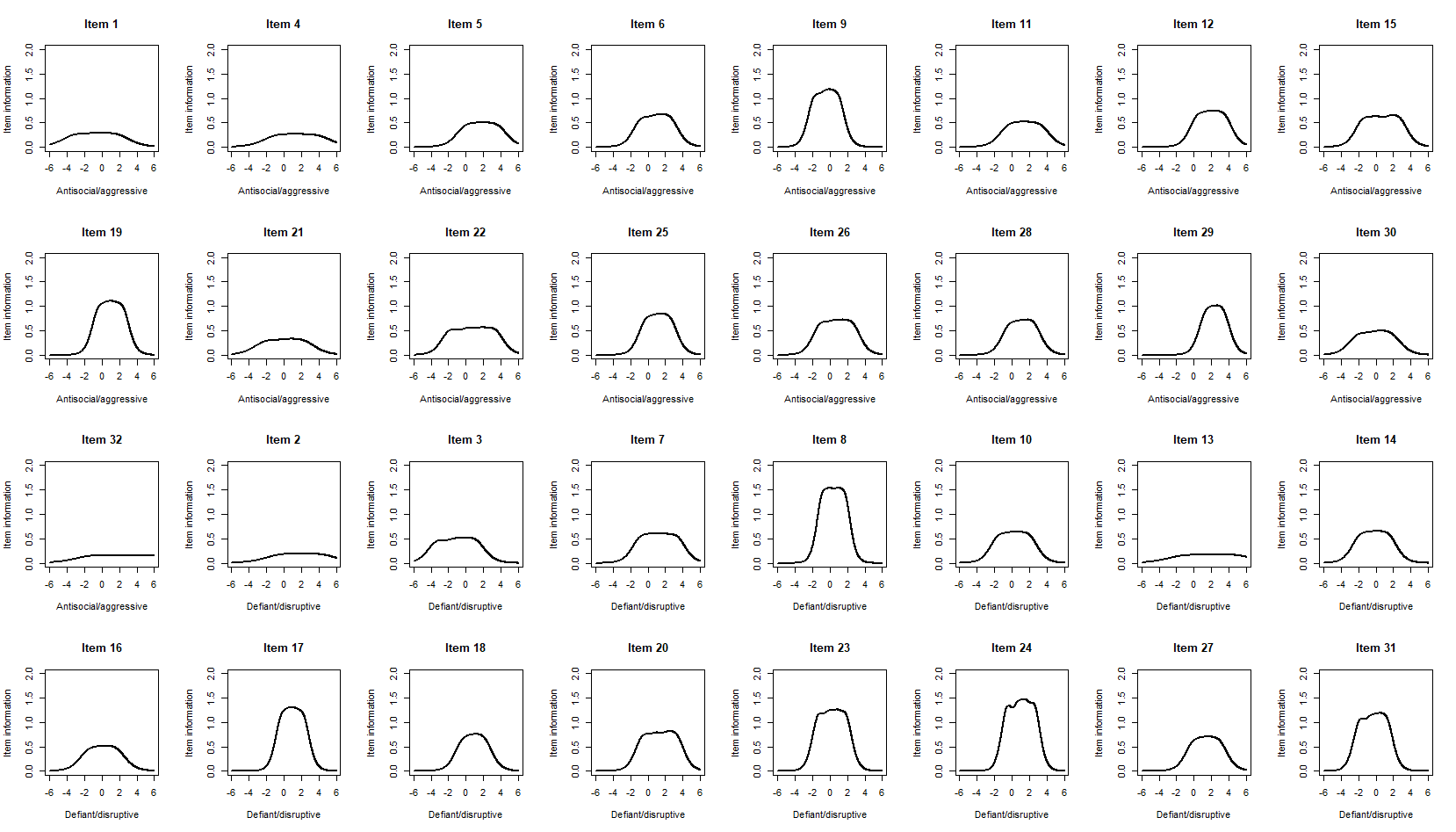
**
